# Development of an adjustable dynamic phantom for testing deviceless motion correction algorithm in PET

**DOI:** 10.1002/mp.70577

**Published:** 2026-07-21

**Authors:** Christian Kühnel, Tabea Nikola Schmidt, Leonie Schreiber, Holger Ginter, Michael Händel, Martin Freesmeyer, Falk Gühne

**Affiliations:** ^1^ Clinic for Nuclear Medicine University Hospital Jena Jena Thuringia Germany; ^2^ Central Research Facilities University Hospital Jena Jena Thuringia Deutschland

## Abstract

**Background:**

Respiratory motion during PET Imaging leads to blurring, volumetric overestimation, and reduced quantitative accuracy. Deviceless motion correction algorithms (MCA) such as OncoFreeze (OF) by Siemens Healthineers (Forchheim, Germany) extract respiratory waveforms from list‐mode data but require validation that cannot be obtained in vivo.

**Purpose:**

To develop and validate an adjustable dynamic phantom with fillable spheres for quantitative assessment of deviceless PET motion correction.

**Methods:**

A microprocessor‐controlled two‐axis phantom was constructed to generate linear craniocaudal (cc), ventrodorsal (vd), and diagonal (diag) motion with amplitudes of 0–40 mm and frequencies of 10–40 min^−^^1^. Polymethylmethacrylate (PMMA) spheres (4, 1, and 0.25 mL) filled with ^1^ ^8^F‐fluorodeoxyglucose (FDG) were placed in a water‐filled PMMA tank and imaged on a Biograph Vision 600 PET/CT (Siemens Healthineers AG, Forchheim, Germany). For each sphere and direction, 2‐min PET scans were acquired with and without MCA, yielding 288 datasets. Volumes, activities, and recovery coefficients (RCs) were extracted using two different isocontours.

**Results:**

Without correction, motion produced substantial overestimation of apparent volume: +15%–55% (4 mL), +81%–166% (1 mL) and +276%–509% (0.25 mL), depending on trajectory and IC_37_. MCA reduced these distortions: For the 1 mL sphere from +87% to −8% (vd) and +166% to +82% (diag). IC_0.1_‐based activity remained within ≈1%–2% of the nominal value and was minimally affected by MCA, while IC_37_ underestimated activity (∼45%) and was direction‐dependently altered by correction. RCs increased with sphere size and were partially improved by MCA but not restored to static levels.

**Conclusions:**

The phantom provides a reproducible ground‐truth framework for evaluating deviceless PET motion correction. MCA reduces motion‐induced blurring and volumetric inflation, though correction remains size‐ and direction‐dependent. The system enables systematic benchmarking and further optimization of motion‐correction algorithms.

## 1 INTRODUCTION

Respiratory motion is a major source of quantitative inaccuracy in PET imaging, particularly in lesions of the chest or upper abdomen. Long acquisition times introduce blurring, volumetric overestimation, and reduced recovery coefficients. These effects directly impact staging accuracy in lung cancer, one of the leading causes of cancer mortality worldwide, and contribute to diagnostic uncertainty in metabolic imaging procedures.^1^, ^2^, ^3^ Despite the high diagnostic value of ^1^ ^8^F‐FDG PET/CT in thoracic oncology,^4^ respiratory motion remains a substantial challenge for image quality and quantification.^5^, ^6^

Deviceless motion correction algorithms (MCA), such as Siemens OncoFreeze (OF), attempt to extract respiratory waveforms directly from PET list‐mode data and reconstruct motion‐compensated images without external tracking hardware.^7^ This also known as elastic motion deblurring (EMDB) algorithms have attracted growing interest as a promising alternative to conventional gating‐based techniques. They avoid the inherent data loss associated with respiratory gating by utilizing up to 100% of the acquired PET data. Initial independent evaluations have relied on ground truth phantom experiments to objectively quantify motion correction performance.^8^ However, these studies required dedicated and cost‐intensive auxiliary equipment, including external respiratory gating systems, which are not routinely available in clinical settings. This dependency on specialized hardware limits the broader applicability and transferability of such validation approaches to standard clinical workflows. Although these approaches offer practical advantages in clinical routine, their quantitative performance requires systematic validation against known ground‐truth motion trajectories, activity distributions, and geometric conditions that are notoriously difficult to obtain in vivo.

In nuclear medicine, phantoms constitute a standard tool for the assessment of diverse quality parameters associated with a range of imaging technologies.^9^ The utilization of phantoms, by simulating anatomical structures and radioactive distributions, facilitates the measurement of activity allocation, resolution limits and the determination of recovery coefficients.^10^, ^11^ As these parameters are of particular significance for the evaluation of image quality and diagnostic accuracy, investigations focusing on the utilization of phantoms in this field represent a research domain of considerable importance. In particular, the use of rapidly prototyped, dynamic, and customizable yet reproducible phantoms remains a field of investigation.^12^

Dynamic phantoms were established in medical physics as a controlled environment for such evaluations and research questions.^13^, ^14^, ^15^ In the field of motion detection, existing systems are frequently limited by fixed trajectories, restricted configurability, or material properties that reduce their suitability for reproducible benchmarking. In particular, there is a lack of low‐cost, programmable phantoms capable of simulating multiple motion directions with fillable radioactive targets under precisely defined conditions.

The objective of this work is to provide a reproducible and technically transparent framework for evaluating deviceless PET motion correction. The presented phantom design and validation establish a methodological foundation for future optimization and standardization of motion‐correction techniques.

## 2 METHODS AND MATERIALS

### 2.1 Phantom design and setup

A motion phantom should meet methodological requirements for controlled imaging experiments involving object displacement.

The primary requirements included:

- the capability to generate reproducible, programmable translational motion in multiple spatial directions
- the use of materials with attenuation properties representative of soft tissue
- the avoidance of metal‐induced imaging artifacts within the field of view.

To address these requirements, the phantom was constructed using polymer‐based materials, primarily polymethyl methacrylate (PMMA) and polyvinyl chloride (PVC), in combination with a water‐filled volume to approximate soft‐tissue attenuation characteristics. Interchangeable hollow inserts filled with radioactive tracer were used to represent focal activity distributions subjected to motion.

Object motion was realized using a motor‐driven linear motion system based on stepping motors, allowing user‐defined motion amplitudes and frequencies. Motion control was implemented via a microcontroller‐based platform with custom firmware enabling the specification of motion direction, timing, and repetition. The motion system was designed to operate independently of the imaging system and to allow reproducible execution of predefined motion patterns.

All components located within the imaging field of view were restricted to non‐metallic materials to comply with the requirement of artifact‐free imaging and consistent attenuation behavior across imaging modalities. Due to the currently used drive components and the microcontroller system, the phantom in its present configuration was not designed for PET/MRI applications.

### 2.2 Procedure

The spheres were filled with predefined activity concentrations between 0.25 and 2 MBq/mL. To standardize preparation and minimize radiation exposure, a stock solution was prepared for each measurement block and distributed into the spheres using sterile syringes. Air bubbles were prevented by filling the spheres carefully. Each sphere's activity was measured in a dose calibrator individually before imaging (ISOMED 2010, NuviaTech Instruments, Dresden, Germany).

### 2.3 Data acquisition

Four different movement patterns were examined: a static one, a unidirectional one in a hypothetical ventrodorsal direction (vd), a unidirectional one in a hypothetical craniocaudal direction (cc), and a bidirectional one in a diagonal direction (diag). Motion parameters were constantly for each movement pattern (vd: 20 mm; cc: 20 mm; diag.: 20 mm VD + 20 mm CC, corresponding to a total path length of ∼28.3 mm) and frequency 16 min^−^^1^ as a proof of concept.^16^ All measurements were performed on a Biograph Vision 600 PET/CT system (Siemens Healthineers, Forchheim, Germany) with 440 × 440 matrix. For each sphere and each motion condition (static, vd, cc, diag), a 2‐min PET acquisition was obtained, according to clinical standard. CT for anatomical reference and attenuation correction was performed in static conditions of the phantom to ensure a comparable reference for each measurement as a CT in motion would have registered the position of the sphere in one random position

A complete measurement series was performed for each sphere and then repeated after one physical half‐life of ^18^F (109.73 min). This procedure continued until approximately one‐eighth of the initial activity remained, yielding three repeated measurement series per sphere. Each series included PET acquisitions for all four motion directions, resulting in a total of 288 reconstructed datasets.

Each dataset was reconstructed once by using MCA and once without using MCA in analogy to the clinical routine.

### 2.4 Data analysis

Image evaluation was performed using the research software PMOD (v4.01. PMOD Technologies, Brucker, Billerica, Massachusetts, USA). PET datasets were fused with their corresponding CT images to visualize geometric agreement and motion‐induced distortions. For quantitative analysis, two segmentation strategies were applied^17^:

- relative isocontour (IC_XX_): a threshold of “x” % of the maximum voxel value, derived from static reference scans, compared with the CT conditions was used as a fixed relative isocontour for activity and volume estimation.
- low threshold isocontour (IC_0.1_): a 0.1 % threshold was used as an inclusive contour capturing nearly all detectable activity, serving as a proxy for total activity independent of partial volume effects.

For each dataset segmented volume, total activity, and recovery coefficient (RC) were extracted. Data were exported from PMOD and further descriptively analyzed using R (version 4.4.1, Posit, Boston, MA, USA). Means, standard deviations, medians, and ranges were reported for continuous variables. Differences between paired datasets were evaluated using a two‐sided paired Student's *t*‐test, with *p* values < 0.05 considered statistically significant.

## 3 RESULTS

### 3.1 Phantom

**FIGURE 1:**
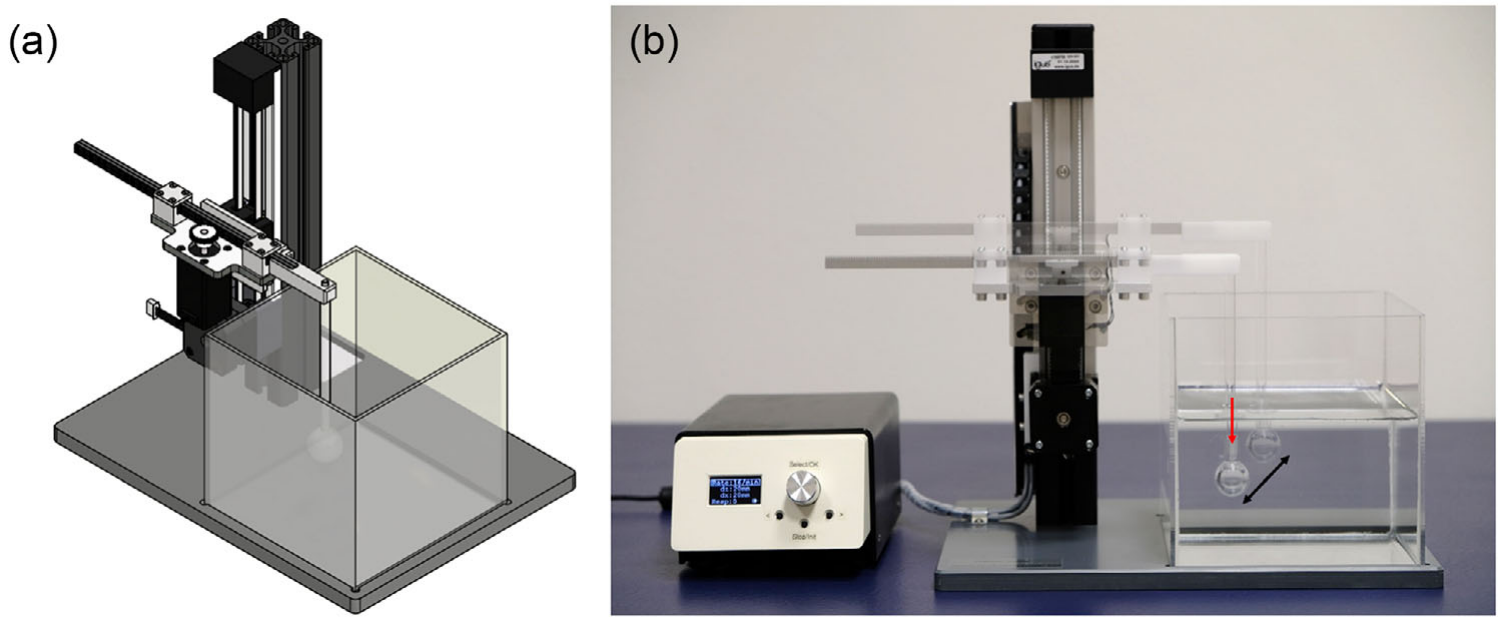
CAD model of the programmable two‐axis dynamic phantom with PMMA water tank and adjustable sphere holder (a). Assembled system consisting of the two‐axis linear drive, stepping‐motor controller, and the external control unit (b). The fillable hollow sphere (red arrow) is positioned inside the water‐filled PMMA tank to provide tissue‐equivalent attenuation. The system allows programmable caudocranial, ventrodorsal, and diagonal motion (black arrow) with adjustable amplitudes and respiratory frequencies for quantitative evaluation of deviceless PET motion correction.

The phantom was designed and constructed at the Central Research Facilities of Jena University Hospital using AutoDesk Inventor Professional 2022 (Autodesk, San Rafael, CA, USA). It consists of a cubic PMMA tank (14.5 × 14.5 × 14.5 cm^3^) filled with water to reproduce soft‐tissue‐equivalent attenuation (Figure 1a). A non‐metallic PMMA holder accommodates hollow spheres of 4 mL (19.8 mm), 1 mL (12.4 mm), and 0.25 mL (7.9 mm) volume (inner diameter) (Data Spectrum Corporation, Durham, NC, USA), which can be filled with ^1^ ^8^F‐FDG to simulate moving hot lesions.

#### 3.1.1 Microprocessor control unit

The mechanical motion system incorporates a two‐axis linear drive unit enabling translational motion in craniocaudal (cc), ventrodorsal (vd), and diagonal (diag) directions. Motion amplitudes of 0–40 mm and respiratory frequencies of 10–40 min^−^^1^ can be programmed (Figure 1b). The drive consists of a JogMode stepping‐motor control unit (igus GmbH, Cologne, Germany) and an Arduino Mega 2560 Pro microcontroller (Joy‐it, SIMAC Electronics GmbH, Neukirchen‐Vluyn, Germany). Custom firmware written in C++ controls the stepping‐motor timing, range limits, acceleration profiles, and user‐selectable motion patterns. The operator can initiate, halt, or reposition the sphere within its mechanical bounds at any time.

The control of the breathing or motion dependent movement of the phantom was implemented on a microcontroller platform using a dedicated firmware written in C++. The program structure follows a modular concept with compiler definitions, macros, and global variables providing the basis for task execution. Initialization routines (setup) configure serial communication for debugging, general purpose input/output (GPIO) pins, electrically erasable programmable read‐only memory (EEPROM), and an OLED display, while hardware timers are employed to ensure precise synchronization of motor and encoder control. Two interrupt service routines (ISR) are defined: one for stepper motor control of the phantom's axes (TIMER1) and another for processing the rotary encoder signals (TIMER3), which enables exact tracking of position and movement phases. The main program loop operates a menu‐driven logic, allowing adjustment of parameters such as in‐ and expiration of breath range, rate, and pause times, which are stored in EEPROM to ensure reproducibility across sessions. Dedicated classes (e.g., for button handling and EEPROM access) facilitate robust user interaction. This architecture ensures deterministic motor control and stable operation of the phantom, enabling reproducible respiratory motion patterns for experimental validation (see supplementary material).

### 3.2 Measurements

A total of 288 PET/CT datasets were acquired and analyzed. The greatest agreement regarding the volume of all static spheres was achieved by measurements using a threshold of a mean IC 37 % (IC_37_) within a range of range between 32 and 44. Therefore, this threshold was applied for contouring of the dynamic measurements. The phantom exhibited highly reproducible motion across all programmed amplitudes and trajectories, with no detectable mechanical drift across repeated measurement series.

**FIGURE 2:**
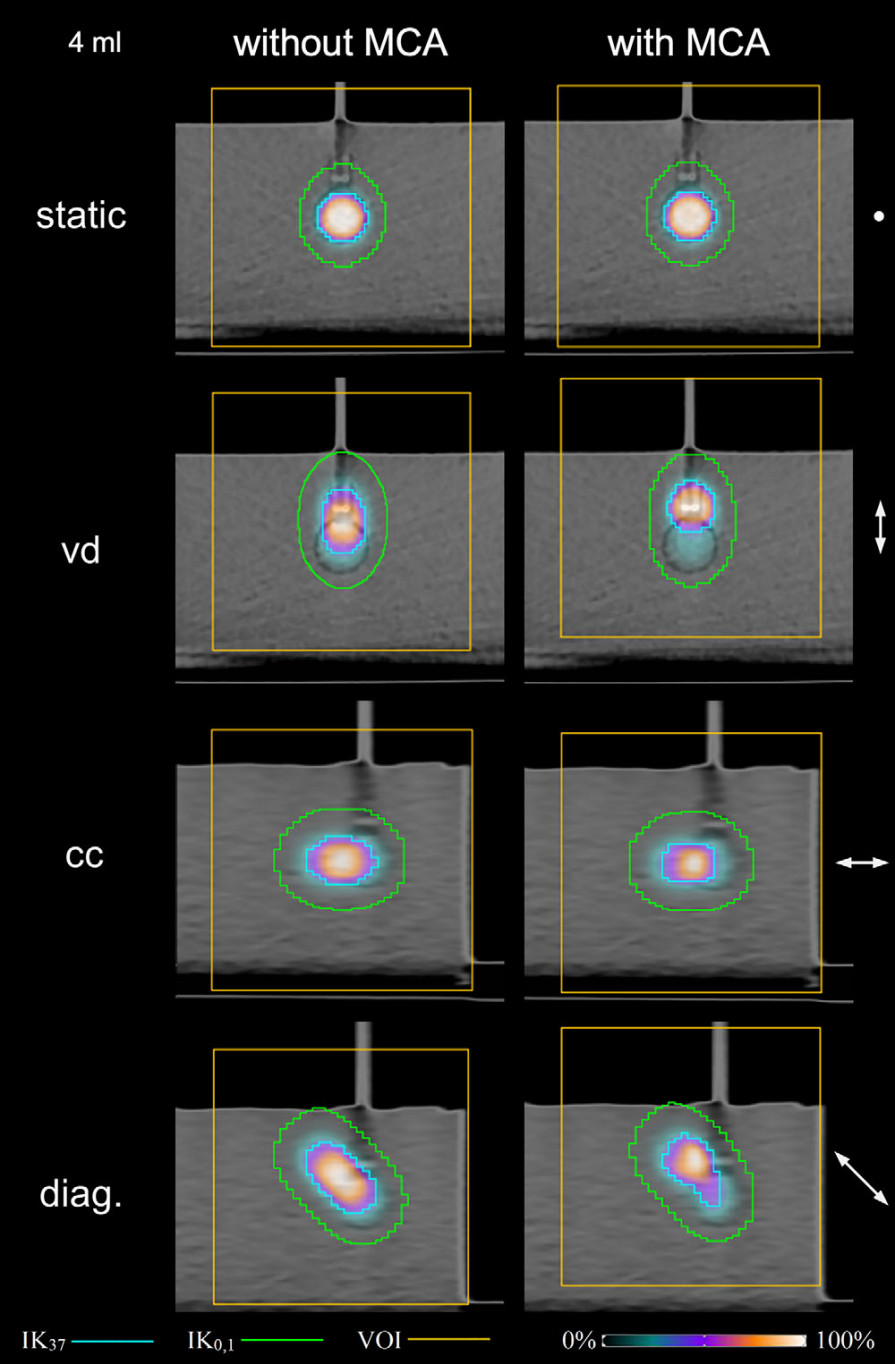
Sagittal PET/CT fusion images of the 4‐ml sphere exemplary for used motion conditions, shown without motion correction (MCA) on the left and with MCA on the right. Rows correspond to the four motion states: static (no motion), ventrodorsal motion (vd), craniocaudal motion (cc), and diagonal motion (diag.). Motion causes characteristic elongation of the activity distribution along the corresponding trajectory, resulting in direction‐dependent shape deformation.

Without motion correction, translational motion produced marked volumetric overestimation and shape elongation, with distortions increasing systematically for smaller spheres and for more complex trajectories (Figure 2). The diagonal direction showed the largest deformation, and the smallest sphere, approaching the spatial resolution limit, exhibited the strongest partial‐volume‐driven exaggeration. These effects corresponded to substantial increases in the apparent principal axis length and were visually consistent with the elongated, elliptical appearance of the moving spheres.

MCA substantially mitigated motion‐induced distortions for all volumes and directions, with the most pronounced improvements observed for ventrodorsal motion. Corrections were less effective for craniocaudal motion and incomplete for the smallest sphere due to dominant partial‐volume effects. In some conditions involving the larger spheres, mild overcorrection was observed. A detailed overview of direction‐ and size‐dependent deviations, before and after correction can be found in Table 1.

**TABLE 1:** Relative mean volumetric distortion (%) for motion directions and sphere sizes, with and without MCA (IC37).

		Isocontour_37_			Isocontour_0.1_		
Sphere volume	Motion direction	Without MCA (%)	With MCA (%)	Correction	Without MCA (%)	With MCA (%)	Correction
4 mL	static	−1	−5	−4	+510	+487	−23
	vd	+15	−16	−31	+784	+696	−88
	cc	+16	−9	−25	+790	+716	−74
	diag	+55	+19	−36	+943	+893	−50
1 mL	static	0	−10	−10	+1150	+1135	−15
	vd	+87	−8	−95	+1956	+1691	−265
	cc	+81	+41	−40	+1994	+1786	−208
	diag	+166	+82	−84	+2456	+2179	−277
0.25 mL	static	+48	+31	−17	+2732	+2812	+80
	vd	+374	+87	−287	+5448	+4072	−1366
	cc	+276	+212	−64	+5416	+6092	+676
	diag	+509	+284	−225	+6808	+5948	−860

Using IC_0.1_, the measured activity concentrations showed close agreement with the nominal filling activities, with deviations remaining within approximately 1%–2% and essentially unaffected by MCA. This finding indicates that the IC_0.1_‐based contouring approach provided robust and reproducible sphere delineation across all measurements. In contrast, IC_37_ consistently underestimated activity concentrations due to partial‐volume effects. MCA influenced IC_37_‐based activity values for moving spheres but had negligible effects on IC_0.1_‐derived measurements.

Statistical analysis demonstrated significant differences between the measurements with and without MCA for most sphere sizes and motion patterns (*p* < 0.001). Only the static measurements of the 0.25 mL spheres did not show a statistically significant difference between both reconstruction approaches (*p* = 0.12098).

**FIGURE 3:**
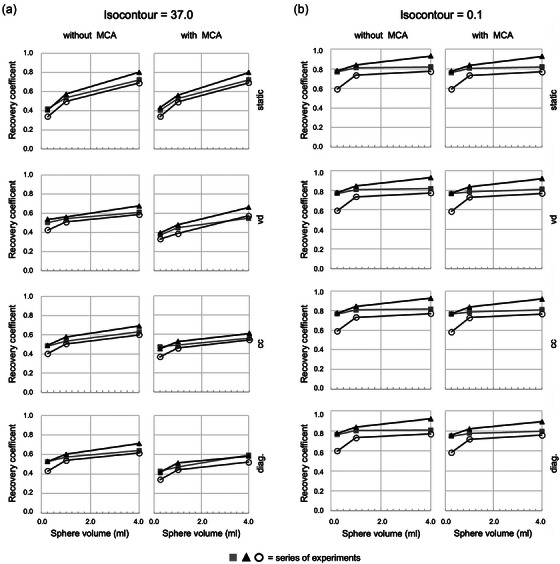
Recovery coefficient (RC) curves for the 4 mL (diameter 19.8 mm), 1 mL (diameter 12.4 mm) and 0.25 mL (diameter 7.9 mm) sphere across all motion conditions for three repeated series. RC values derived from the relative isocontour threshold IC37 with and without motion correction algorithm (MCA) (a). RC values derived from the inclusive low‐threshold contour IC0.1 with and without MCA (b). Each row corresponds to one motion condition: static (no motion), ventrodorsal (vd), craniocaudal (cc), and diagonal (diag). RC increases with sphere volume across all conditions. Motion reduces RC relative to static measurements, and MCA partially restores RC values with direction‐dependent effectiveness. IC37 reflects threshold‐based quantification and demonstrates stronger motion sensitivity, whereas IC0.1 captures nearly all detectable activity and therefore shows higher and more stable RC values.

Recovery coefficients increased with sphere size in accordance with known partial‐volume behavior (Figure 3). The repeated RC measurements demonstrated high reproducibility across all decay‐corrected acquisitions without systematic trends in activity or volume over successive half‐life‐shifted repetitions. MCA reduced discrepancies between static and moving RCs but did not fully restore RCs to static conditions, while only minor quantitative differences were observed between datasets with and without MCA. This indicates that the algorithm did not substantially alter or discard quantitative signal information under the investigated conditions, although correction magnitude remained direction dependent.

## 4 DISCUSSION

This proof‐of‐concept presents a user‐interface‐friendly programmable, low‐cost dynamic phantom designed with a low‐footprint to provide reproducible and easy to use ground‐truth conditions for evaluating deviceless PET respiratory motion correction. The phantom reliably reproduced defined motion patterns and enabled quantitative assessment of the MCA OF algorithm under controlled conditions, without moving the whole setup like in previously presented phantoms like the reciprocating table.^18^ Furthermore, the phantom setup employed by Meier et al. relied on elastic bands and string connections to simulate tumor motion trajectories, a construction that inherently introduces mechanical variability between repeated measurements and therefore limits the reproducibility of the experimental results.^8^ Additionally, cost‐intensive equipment, such as the Anzai AZ‐733V belt sensor (Anzai Medical Co., Ltd.; Tokyo, Japan) as well as dynamic thorax phantoms from CIRS (CIRS Inc., Norfolk, Virginia, USA) could be omitted by the setup presented in this study. The results demonstrate that both the magnitude and direction of motion substantially influence PET quantification, and that deviceless correction can reduce, but not eliminate, motion‐induced blurring and misregistration. Especially Figure 2 shoes in the case of diagonal motion, residual elongation and tailing effects along the motion path may therefore lead to irregular or “comet‐like” segmentations despite the underlying programmed motion being linear in this proof‐of‐concept. Such behavior may reflect interactions between motion correction, interpolation, reconstruction, and partial‐volume effects.

Without motion correction, the phantom showed the expected combination of volumetric overestimation and activity underestimation, reflecting temporal blurring of the radioactive sphere along its trajectory during the acquisition for the mean IC_37_ (range 32 and 44).^19^ This effect has been widely described in the PET literature and is a direct consequence of the finite spatial resolution, positron range, detector sampling, and reconstruction mechanics.^20^, ^21^ The degree of distortion increased markedly for smaller spheres, consistent with partial‐volume effects dominating below ≈ 2–3x the system's full width at half maximum. The data of the 0.25 mL sphere (≈ 7.9 mm diameter) shows a critical range for IC_37_, explaining the extreme apparent volume inflations of + 509 (without MCA) or + 284% (with MCA). These findings align with classical descriptions of PET recovery behaviour.^22^, ^23^ The effect of overestimation of volume is found to be significantly more effective in the case of IC_0.1_ (Table 1). It is also curious that MCA can also result in deterioration in volume measurement für IC_0.1_ motion pattern diag. and vd.

Motion direction emerged as a key determinant of distortion. Diagonal (diag) motion produced the largest volumetric deviations, followed by ventrodorsal (vd) and craniocaudal (cc) motion. This anisotropy reflects both the geometry of PET sampling and variable spatial resolution across planes, with the transverse (vd‐related) direction typically offering higher effective resolution than the axial direction.^24^ The phantom thus reproduces not only motion artifacts but also resolution anisotropies inherent to PET/CT systems.

MCA significantly reduced these distortions for all sphere sizes. The strongest improvements occurred for vd motion, where the deviation for the 1 mL sphere was reduced from +87% to −8%. This confirms that MCA can robustly estimate and correct motion components within transaxial planes, consistent with the algorithm's list‐mode‐based derivation of per‐slice motion signals. In contrast, cc motion was less effectively corrected, and diagonal trajectories showed intermediate behavior. This is particularly disadvantageous, as the cc movement is the one mimicking the natural breathing motion. These results suggest that the MCA OF motion estimation accuracy varies depending on the predominant direction of displacement, which is plausible given the dependence on sampling frequency, voxel geometry, and the stability of respiratory waveform extraction.

Figure 3 illustrates the recovery coefficients (RC) for both IC methods across all motion conditions. For IC_0.1_ (Figure 3b), RC values remain consistently high (∼0.75–0.90) and are largely unaffected by the application of MCA across all sphere sizes, motion directions, and amplitudes. This stability reflects the inherent tolerance of the low absolute threshold to motion‐induced blurring: the IC encompasses nearly the entire activity distribution regardless of spatial smearing, and no counts are effectively excluded by the segmentation. MCA therefore neither substantially improves nor degrades RC performance at IC_0.1_. For IC_37_ (Figure 3a), RC values are considerably lower overall and exhibit a strong dependence on sphere size, consistent with partial volume effects. Notably, in the diagonal motion condition (Figure 3a, bottom row), the application of MCA results in a paradoxical reduction of RC compared to the uncorrected dataset. When objects approach the resolution limit, even perfect motion estimation cannot restore the underlying activity distribution, as confirmed by recovery coefficient theory and prior phantom studies.^22^, ^25^

Activity quantification showed minimal differences for IC_0.1_, indicating that MCA does not alter total detected counts. Threshold‐based parameters (IC_37_), which mimic clinical segmentation, were more sensitive to motion and correction. This has implications for quantitative PET metrics that rely on fixed relative thresholds, such as metabolic tumor volume or response assessment metrics. These results attributed the limitations of the MCA in the case of complex, multidirectional motion, and further studies should investigate this issue, also considering varying positional deviation from the isocenter.

A limitation of the present phantom proof‐of‐concept is the simplified motion, which does not replicate physiological hysteresis, non‐linear trajectories, or inspiratory and expiratory plateaus, as well as the missing background.^5^, ^16^ Nevertheless, the high reproducibility of the platform offers a significant advantage for algorithm benchmarking, enabling controlled comparisons that are impossible in studies at patients.

Overall, the phantom provides a robust, transparent tool for evaluating deviceless PET motion correction and highlights the direction‐ and size‐dependent performance of OF. Future developments could incorporate 3D printed anthropomorphic geometries mimicking different tissues, multi‐lesion configurations, and patient‐derived respiratory traces to further enhance realism end‐expiration respiratory gating and support inter‐algorithm comparisons.^10^, ^26^ The small footprint and modular design of the phantom may additionally facilitate future AI‐based MCA applications in combination with anthropomorphic Alderson phantom components. This AI based feature extract information from the CT topogram and patient positioning within the bed position to adapt the strength of motion correction algorithms according to the expected respiratory motion burden.

## 5 CONCLUSION

This work presents a programmable dynamic phantom that enables reproducible, ground‐truth‐based evaluation of deviceless PET motion correction. The system reliably generates controlled motion trajectories and provides quantitative insight into the direction‐ and size‐dependent performance of the MCA called OF. While OF substantially reduces motion‐induced blurring and volumetric inflation, residual errors remain, particularly for small structures and non‐transverse motion components, reflecting fundamental limits imposed by system resolution and motion‐estimation accuracy.

The results demonstrate the utility of the phantom as a transparent benchmark for validating reconstruction‐based motion correction algorithms. However, the present study is restricted to linear trajectories and a single breathing frequency. To more comprehensively characterize algorithm performance, further studies incorporating a wider range of respiratory rates, non‐linear patient‐derived motion patterns, and more complex anthropomorphic geometries will be essential as well as for different radionuclides and cold spots as well. Such extensions will enable systematic optimization, cross‐algorithm comparison, and improved translation of deviceless motion correction into clinical practice.

## CONFLICT OF INTEREST STATEMENT

The authors have no relevant conflicts of interest to disclose.

## Supporting information



Supporting Information

## Data Availability

The datasets generated and/or analyzed during the current study are available from the corresponding author upon reasonable request.
