# Supplementary material for: Development of an adjustable dynamic phantom for testing deviceless motion correction algorithm in PET
Source: Med Phys. 2026 Jul 21;53(8):e70577. doi: 10.1002/mp.70577 (PMC13389335; doi:10.1002/mp.70577)
Supplement: Supplementary file 1 — Supporting Information [file MP-53-0-s001.pdf]

Flow Chart "Phantom Atmung v1.1"

This is only a coarse overview about the main parts. Not all functions are shown!

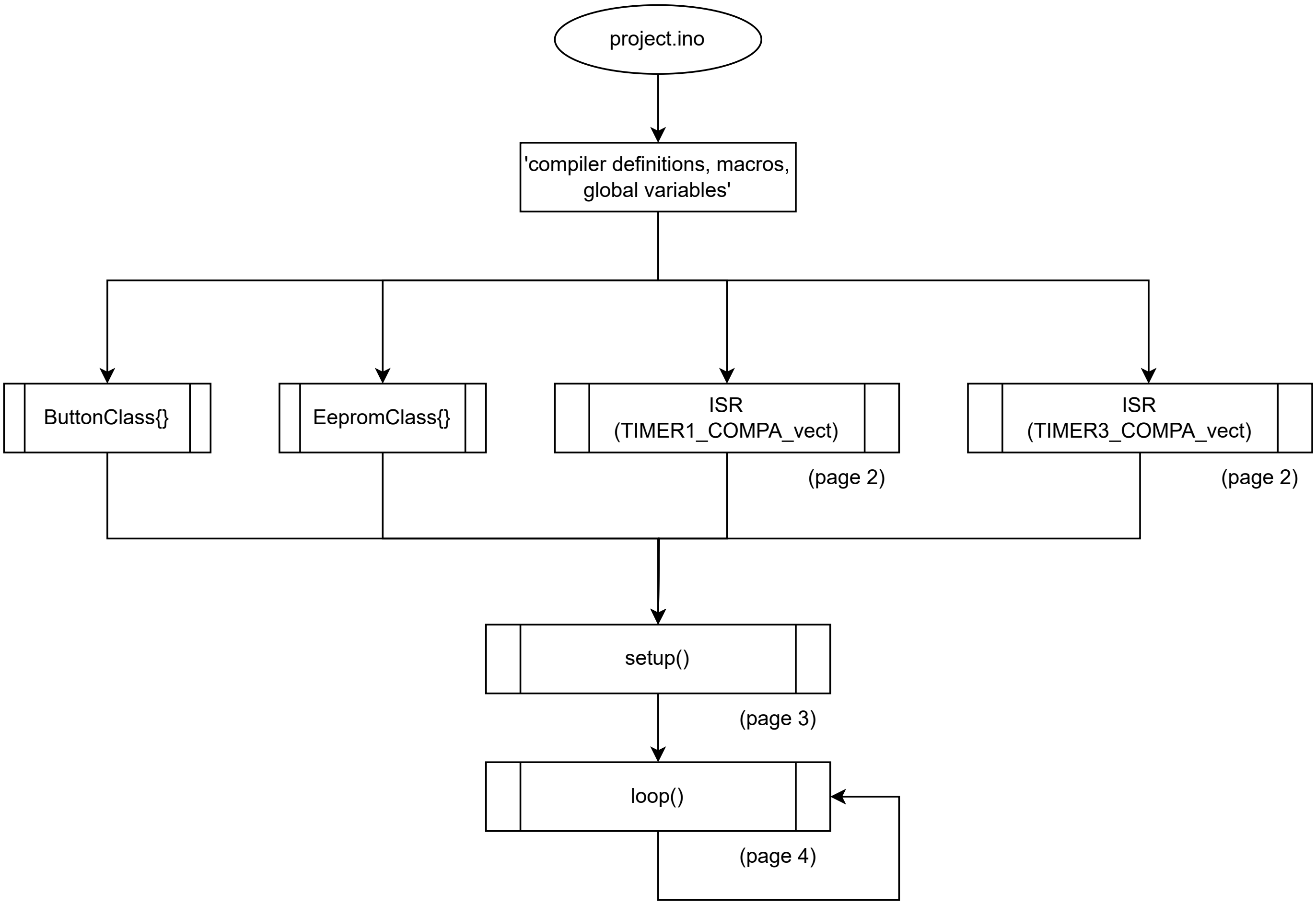

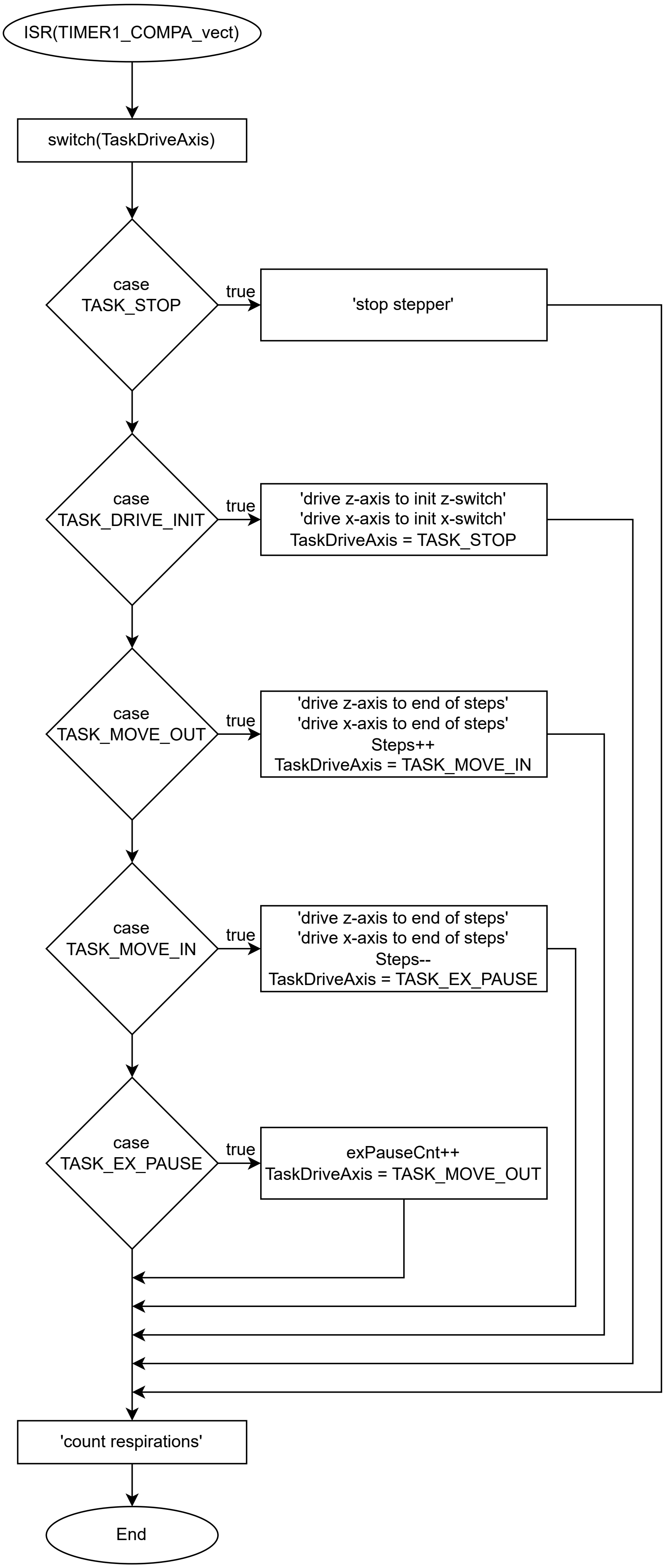
ISR(TIMER1_COMPA_vect)
switch(TaskDriveAxis)
case TASK_STOP
true
'stop stepper'
case TASK_DRIVE_INIT
true
'drive z-axis to init z-switch'
'drive x-axis to init x-switch'
TaskDriveAxis = TASK_STOP
case TASK_MOVE_OUT
true
'drive z-axis to end of steps'
'drive x-axis to end of steps'
Steps++
TaskDriveAxis = TASK_MOVE_IN
case TASK_MOVE_IN
true
'drive z-axis to end of steps'
'drive x-axis to end of steps'
Steps--
TaskDriveAxis = TASK_EX_PAUSE
case TASK_EX_PAUSE
true
exPauseCnt++
TaskDriveAxis = TASK_MOVE_OUT
'count respirations'
End

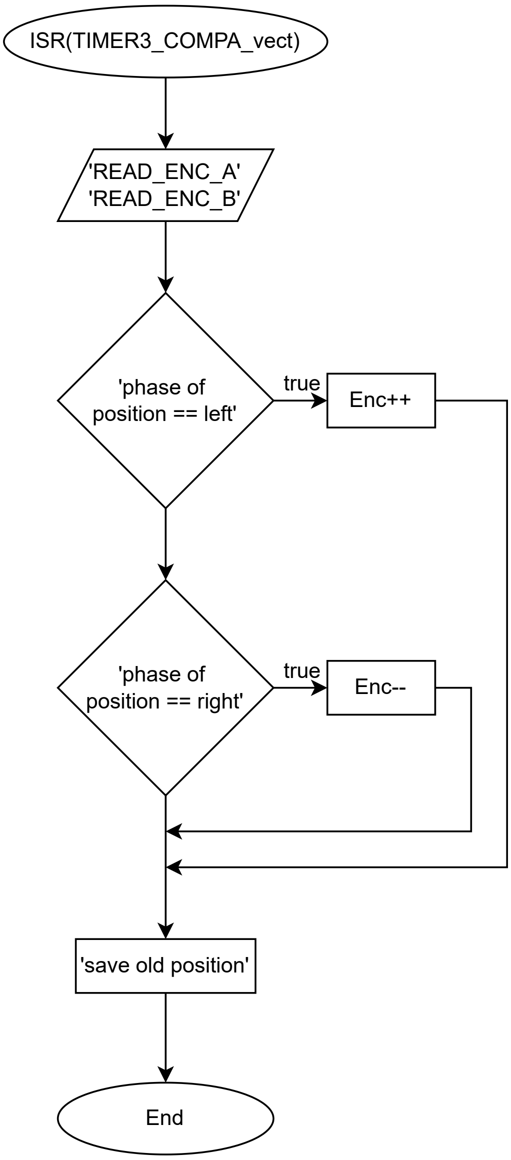
ISR(TIMER3_COMPA_vect)
'READ_ENC_A'
'READ_ENC_B'
'phase of position == left'
true
Enc++
'phase of position == right'
true
Enc--
'save old position'
End

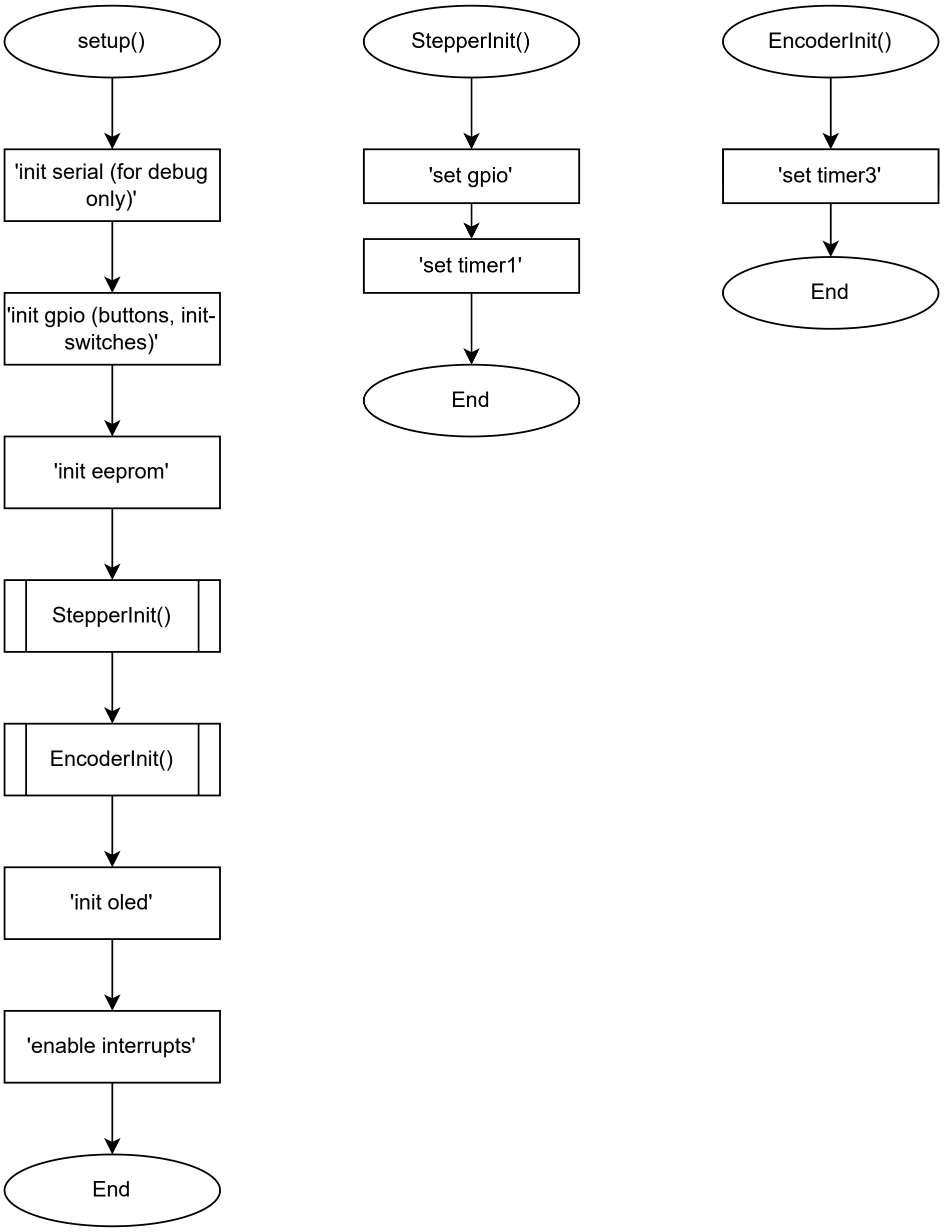
setup()
'init serial (for debug only)'
'init gpio (buttons, init-switches)'
'init eeprom'
StepperInit()
EncoderInit()
'init oled'
'enable interrupts'
End
StepperInit()
'set gpio'
'set timer1'
End
EncoderInit()
'set timer3'
End

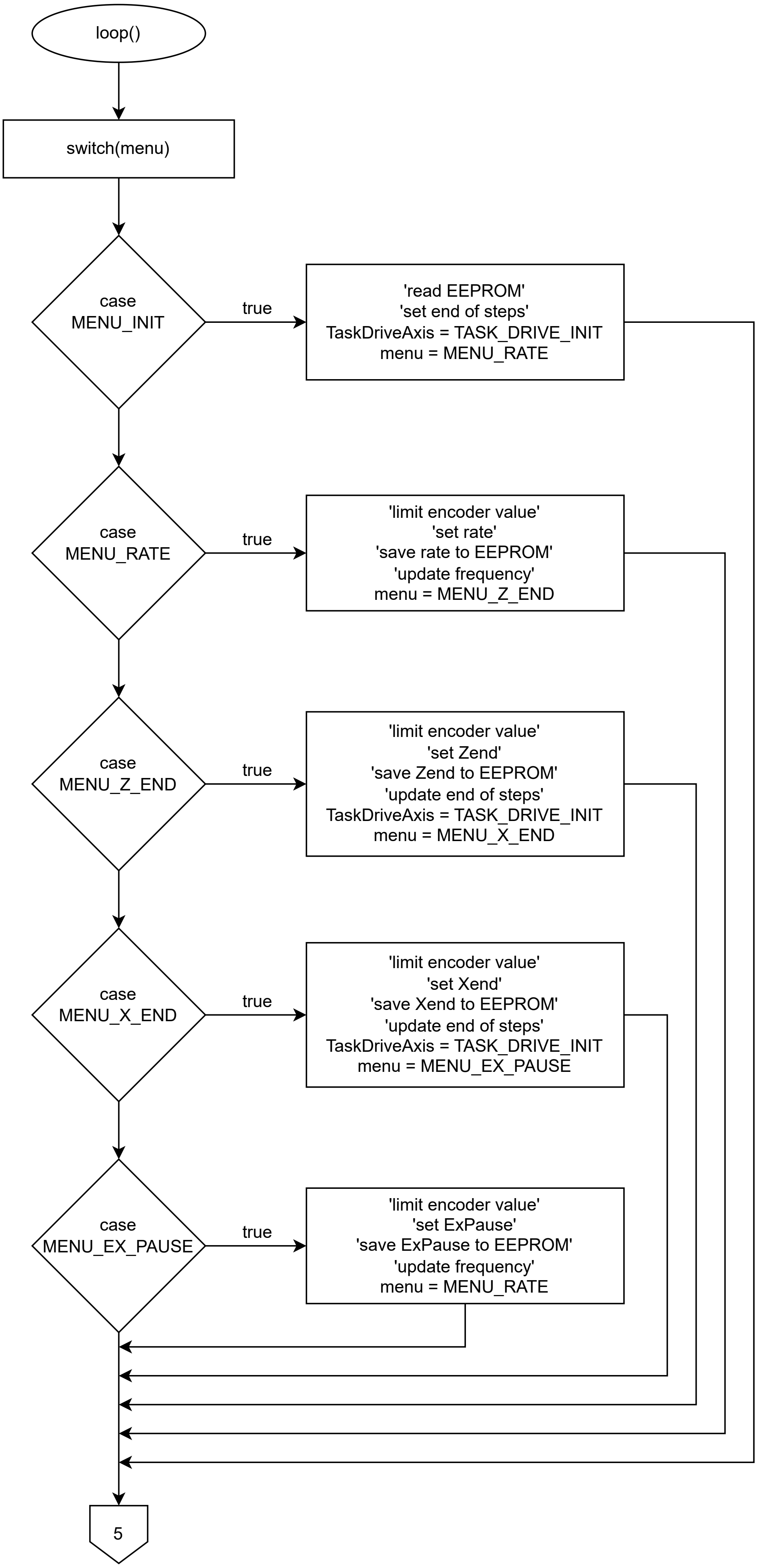
loop()
switch(menu)
case
MENU_INIT
true
'read EEPROM'
'set end of steps'
TaskDriveAxis = TASK_DRIVE_INIT
menu = MENU_RATE
case
MENU_RATE
true
'limit encoder value'
'set rate'
'save rate to EEPROM'
'update frequency'
menu = MENU_Z_END
case
MENU_Z_END
true
'limit encoder value'
'set Zend'
'save Zend to EEPROM'
'update end of steps'
TaskDriveAxis = TASK_DRIVE_INIT
menu = MENU_X_END
case
MENU_X_END
true
'limit encoder value'
'set Xend'
'save Xend to EEPROM'
'update end of steps'
TaskDriveAxis = TASK_DRIVE_INIT
menu = MENU_EX_PAUSE
case
MENU_EX_PAUSE
true
'limit encoder value'
'set ExPause'
'save ExPause to EEPROM'
'update frequency'
menu = MENU_RATE
5

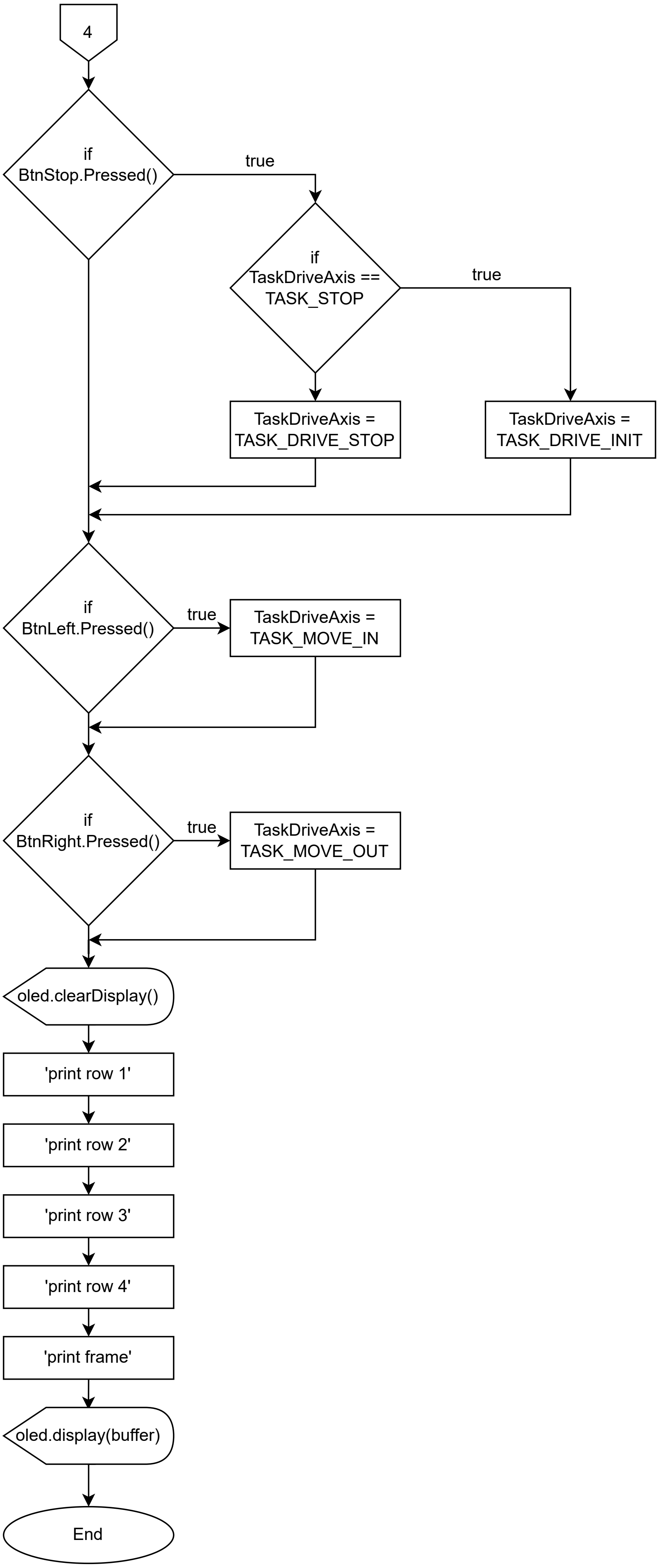
4
if BtnStop.Pressed()
true
if TaskDriveAxis == TASK_STOP
true
TaskDriveAxis = TASK_DRIVE_STOP
TaskDriveAxis = TASK_DRIVE_INIT
if BtnLeft.Pressed()
true
TaskDriveAxis = TASK_MOVE_IN
if BtnRight.Pressed()
true
TaskDriveAxis = TASK_MOVE_OUT
oled.clearDisplay()
'print row 1'
'print row 2'
'print row 3'
'print row 4'
'print frame'
oled.display(buffer)
End
